# The cuticle and plant defense to pathogens

**DOI:** 10.3389/fpls.2014.00274

**Published:** 2014-06-13

**Authors:** Mario Serrano, Fania Coluccia, Martha Torres, Floriane L’Haridon, Jean-Pierre Métraux

**Affiliations:** Department of Biology, University of FribourgFribourg, Switzerland

**Keywords:** *Arabidopsis*, innate immunity, *Botrytis cinerea*, resistance, cuticle, cutin monomers, wax, ROS

## Abstract

The cuticle provides a physical barrier against water loss and protects against irradiation, xenobiotics, and pathogens. Components of the cuticle are perceived by invading fungi and activate developmental processes during pathogenesis. In addition, cuticle alterations of various types induce a syndrome of reactions that often results in resistance to necrotrophs. This article reviews the current knowledge on the role of the cuticle in relation to the perception of pathogens and activation of defenses.

## INTRODUCTION

The cuticle is a barrier coating the outer surface of epidermal cells of organs of the aerial parts of the plants. It protects against water loss, various abiotic and biotic stress. The structure and properties of the cuticle has received increased attention in the past years and a number of detailed reviews have been published (Martin, 1964; Kolattukudy, 1985; Goodwin and Jenks, 2005; Muller and Riederer, 2005; Reina-Pinto and Yephremov, 2009a; Schreiber, 2010; Domínguez et al., 2011a,b; Nawrath et al., 2013; Yeats and Rose, 2013). The cuticle is structurally diverse among species but exhibits the organization of a composite material consisting in cutin, a polyester that is partly covered and interspersed with waxes (epicuticular and intracuticular waxes). The epicuticular waxes and the cuticle with intracuticular waxes are referred to as the cuticle proper. The cuticle proper lies above a so-called cuticular layer made of cutin and polysaccharides that is closely associated with the cell wall of the underlying epidermis cell. The cutin polymer is typically made of esterified ω- and mid-chain hydroxy and epoxy C16 and C18 fatty acids and some glycerol (Heredia, 2003). This polymer can be cleaved by esterases and yields various cutin monomers. The cuticular wax, is a complex mixture of very long-chain fatty acids (C20–C40) and their derivatives that include alkanes, aldehydes, primary and secondary alcohols, ketones, and esters. Depending on the species, secondary metabolites, such as flavonoids and triterpenoids are also found among the wax components (Samuels et al., 2008). An increasing number of genes involved in the biosynthesis of the cuticle have been identified mainly in *Arabidopsis thaliana* and help to understand its biosynthesis (Pollard et al., 2008; Kunst and Samuels, 2009; Beisson et al., 2012; Bernard and Joubès, 2013; Lee and Suh, 2013). The overall picture of cutin synthesis whereby precursors are assembled in the cell and exported to the cell wall can now be completed but many details still remain unanswered; for example, the nature of the exported cutin or wax precursors, the process of extracellular assembly or the elements involved general control of this complex developmental process. Highlights of the advances in this area comprise the identification of an ABC transporter ABCG32/PEC1 involved in cuticle assembly (Bessire et al., 2011), the description of several classes of transcription factors involved in cutin and wax biosynthesis (Javelle et al., 2010; Seo et al., 2011; Nadakuduti et al., 2012) or post-transcriptional regulation of cuticle biosynthesis by the zinc-finger protein SERRATE (Voisin et al., 2009). The involvement of protein monoubiquitination in the regulation of cuticle biosynthesis was recently documented as several genes of cutin and wax biosynthetic pathway were found to be targets for histone H2B monoubiquitination (Ménard et al., 2014).

Here we will focus on the function of the plant cuticle in relation to the interaction with leaf pathogens.

## THE CUTICLE AS A SOURCE OF SIGNALS

A number of recent reviews have been published that describe various aspects of the biological functions of the cuticle in relation to their physical and biochemical properties (Muller and Riederer, 2005; Reina-Pinto and Yephremov, 2009a). The focus of this chapter will be dedicated to the hypothesis that the cuticle might constitute a potential source of signals for the pathogens or for the plant itself.

### PERCEPTION OF CUTICLE COMPONENTS BY FUNGI

Cutin hydrolysates were shown early on to induce the activity of an extracellular cutinase in *Fusarium solani pv*. *pisi*. Fractionation of the cutin hydrolysates established that the ω-hydroxy fatty acid fraction contained most of the activity. The optimal length of the aliphatic chain is 16 carbons, the activity mostly depends on a hydroxyl group at the ω carbon whereas the presence of the carboxyl group had no significant effect (Lin and Kolattukudy, 1978). Chemically synthesized cuticle monomers also activate fungal development (Ahmed et al., 2003). Kolattukudy (1985) proposed that cuticle-degrading pathogens sense plant surfaces by cutin monomers that activate fungal cutinolytic activity. Cutin monomers are initially generated by basal cutinase activity in fungal spores landing on plant surfaces. Sensing of cutin monomers would then induce high levels of cutinase required for penetration. The induction of cutinase in *F. oxysporum* results from a transcriptional activation (Woloshuk and Kolattukudy, 1986). Furthermore, a transcription factor CTF1 was identified that binds to a G-rich palindromic binding site of the cutinase promoter (Kamper et al., 1994). Cuticular components can also induce other aspects of fungal developmental. For example, cutin monomers induce the germination and appressorium in the rice blast fungus *Magnaporthe grisea* (Gilbert et al., 1996); and appressorial tube formation in *Erysiphe graminis* (Francis et al., 1996). Cutin monomers also induce a protein kinase, LIPK (lipid-induced protein kinase) in *Colletotrichum trifolii*, the causal agent of alfalfa anthracnose. LIPK is essential for triggering infection structure formation in the fungus (Dickman et al., 2003). Besides cutin monomers, surface waxes also activate development processes in fungi. For instance, surface waxes of avocado, including terpenoid components, induce germination and appressorium formation in *C. gloeosporioides*, a pathogen of avocado, while waxes from other plants were not effective (Podila et al., 1993; Kolattukudy et al., 1995). Chloroform extracts of wax from wheat leaf surfaces induce appressorium in *Puccinia graminis* f.sp. *tritici* (Reisige et al., 2006). Appressorium formation in the rice pathogen *M. grisea* is induced by leaf wax of rice or other plants or synthetic n-C22 fatty acid, fatty alcohol or alkane (Hegde and Kolattukudy, 1997). Recently, it was shown that the pre-penetration processes of the powdery mildew fungus *Blumeria graminis* f. sp. *hordei* is stimulated by very-long-chain aldehydes that are wax constituents of the cuticle (Ringelmann et al., 2009; Hansjakob et al., 2010, 2011). For example, during the formation of the primary germ tube in *Blumerica graminis* f.sp. *hordei*, very-long-chain aldehydes (typical components of surface waxes) can stimulate the migration of the nucleus inside the conidia toward the site of primary germ tube emergence (Hansjakob et al., 2012).

Taken together, these observations document the perception of cuticular components by fungi. In the next section, we will show that the plant itself can also detect and react to components of the cuticle.

### THE CUTICLE AND THE PERCEPTION OF ITS PRODUCTS BY THE PLANT

The action of fungal cutinase and related enzymes during the early stages of fungal contact with plant surfaces prepares the infection site both for adhesion and penetration (Deising et al., 1992; Nielsen et al., 2000). Cuticle breakdown products constitute potential signals perceived by the plant that are among the first elicitors to be generated during infection. While it is difficult to determine the nature and concentration of cutin monomers at the infection court, the hypothesis that such monomers could be perceived by the plant was tested in barley and rice treated by ectopic treatments with synthetic analogs (Schweizer et al., 1994, 1996b). Two monomers of the C18 family were effective in protecting barley against *E. graminis* and rice against *M. grisea*, most likely by acting on the plant since these molecules have no direct fungicidal effect. Treatment of suspension-cultured potato cells with cutin monomers induces medium alkalinization, production of ethylene (ET) and accumulation of defense-related genes (Schweizer et al., 1996a). The most active compound was n,16-hydroxypalmitic acid (*n* = 8, 9, or 10), a predominant component of the potato cuticle. When etiolated cucumber hypocotyls are gently abraded, cutin monomers from hydrolysates of cucumber, apple, and tomato cutin induce the production of H_2_O_2_ (Fauth et al., 1998). The gentle abrasion was proposed to reproduce the action of cutinase released by a potential pathogen allowing the plant to perceive and respond to cutin monomers that can readily diffuse through the permeabilized cuticle. A surprising observation of the action of cutinase was made by the addition of purified cutinase from *Venturia inaequalis* or from *F. solani* directly to spores of *Rhizoctonia solani* prior to inoculation of bean leaves. A decrease in symptoms was observed in inoculation droplets containing spores together with cutinase compared to spores with water. The effect of cutinase depends on its lipolytic esterase activity. Pathogenesis-related (PR) protein genes were not associated with cutinase-induced resistance responses of bean leaves in response to cutinase action (Parker and Koller, 1998). This intriguing observation was pursued further by directly expressing a fungal cutinase gene in the cell wall of plants. To this purpose, a cutinase gene from *F. solani pv*. *pisi* was expressed in *Arabidopsis thaliana* under the control of the CaMV35S promoter and targeted to the cell wall (Sieber et al., 2000). A normal layer of wax, but a partly absent cuticle, characterizes cutinase-expressing plants that exhibit enhanced permeability to solutes. A subsequent study provided a detailed assessment to the reaction toward pathogens (Chassot et al., 2007). No difference was observed between cutinase-expressing plants (so-called CUTE plants) and wild types after infection with the biotrophs *E. cichoracearum*, *Hyaloperonospora parasitica,* and *Phytophthora brassicae* or the non-host *Blumeria graminis*. Importantly, CUTE plants displayed almost complete immunity toward the necrotrophic fungus *Botrytis cinerea*. The protection requires the enzymatic activity of the protein, since transformants with a cutinase gene mutated in the active site of the enzyme are not protected. Ectopic application of *Fusarium* cutinase to *Arabidopsis thaliana* leaves also protects against *Botrytis cinerea* and is not the result of a direct action of the cutinase on *Botrytis cinerea,* in agreement with the overexpression experiments (Chassot et al., 2007). Expression of the lipase A gene of *Botrytis cinerea* also provides full protection, confirming the importance of the cutinolytic activity for protection (Chassot et al., 2007). To some extent this is reminiscent of the experiments of Parker and Koller (1998) where active cutinase mixed to spores of *R. solani* led to protection in bean leaves. There was no correlation between the expression of marker genes for the salicylic acid (SA), ET, or jasmonic acid (JA) pathways and expression of the cutinase gene of *F. solani* in *Arabidopsis thaliana* mutants of the SA (*pad4, sid2*), ET (*etr1, ein2, pad2*) and of the JA (*jar1*) pathways clearly show fully independence of cutinase-induced protection on SA, ET, and JA. A number of genes identified from microarray experiments showed an earlier and stronger expression after inoculation with *Botrytis cinerea* of CUTE plants compared to wild types. Fifteen genes were selected and overexpressed in *Arabidopsis thaliana* and eight of these provided increased tolerance to *Botrytis cinerea*. These genes included members of the lipid transfer protein (LTP), the peroxidase (PO), and the protein inhibitor (PI) gene families. Members of the LTPs, PER, and PIs could each contribute in part to the observed resistance induced by *Botrytis cinerea* in CUTE plants (see discussion in Chassot et al., 2007, 2008). Resistance in CUTE plants was also proposed to result from the rapid diffusion of a potential fungitoxic metabolite through the permeable cuticular layer into the inoculation droplet. A fungitoxic activity was observed in the inoculation droplets of *Botrytis cinerea* spore suspension placed on CUTE but not on wild type plants (Chassot et al., 2007) but the chemical nature of the leaf diffusate has not yet been characterized.

A number of studies have reported on *Arabidopsis thaliana* mutants impaired in various aspects of the biosynthesis of the cuticle or that have otherwise an increase in cuticular permeability. An intriguing observation is that several but not all cuticle mutants have an altered permeability and an increased resistance to *Botrytis cinerea* (**Table 1**).

**Table 1:** Mutants displaying alterations in the cuticle structure or in permeability.

Mutant	Plant	Function of wild type gene product	Properties			
			Resistance to *B. cinerea*	Cuticle permeability	Fungitoxic diffusate	
*lcr, lacerata*	*A.t.*	CYP86AB catalyzes w-hydroxylation of fatty acids ranging from C12 to C18:1	+	+	nt	Wellesen et al. (2001), Bessire et al. (2007)
*Hth, hothead; allelic to adhesion of calyx edges (ace)*	*A.t.*	Protein with sequence similarity to long-chain FA w-alcohol dehydrogenases	±	±	nt	Lolle et al. (1998), Kurdyukov et al. (2006a), Bessire et al. (2007)
*Bdg, bodyguard*	*A.t.*	Member of the a/b-hydrolase fold protein superfamily	+	+	+	Kurdyukov et al. (2006b), Chassot et al. (2007)
*lacs2.3, long-chain acyl-CoA synthetase*	*A.t.*	Long-chain acyl-CoA synthetase	+	+	+	Bessire et al. (2007)
*sma4, symptoms to multiple avr genotypes4*	*A.t.*	Long-chain acyl-CoA synthetase2	+	+	+	Tang et al. (2007)
*fdh, fiddlehead*	*A.t.*	Likely to be involved in the synthesis of long chain fatty acids	+	nt	nt	Yephremov et al. (1999), Pruitt et al. (2000), Voisin et al. (2009)
*pec1, permeable cuticle1*	*A.t.*	ATP binding cassette 32 (ABCG32) transporter	+	+	+	Bessire et al. (2011)
*myb96, myeloblastosis transcription factor 96*	*A.t.*	ABA-responsive R2R3 type transcription factor	+	+	nt	Seo et al. (2011), Benikhlef et al. (2013)
*sitiens*	*S.l.*	Abscisic aldehyde oxidase	+	+	nt	Curvers et al. (2010)
*aba2, ABA biosynthesis*	*A.t.*	Short-chain alcohol dehydrogenase	+	+	nt	Cheng et al. (2002), L’Haridon et al. (2011)
*aba3, ABA biosynthesis*	*A.t.*	MoCo sulfurase	+	+	nt	Bittner et al. (2001), Xiong et al. (2001), L’Haridon et al. (2011)

*A.t., Arabidopsis thaliana; S.l., Solanum lycopersicum; nt, not tested.*

The *lcr (lacerata*) mutant is impaired in a gene coding for a cytochrome P450 monooxygenase involved in the formation of ω-hydroxy fatty acids in yeast and could be involved in cutin biosynthesis (Wellesen et al., 2001). Reduced levels of the major constituents of cuticular polyesters and cutin were observed in the *hth* (allelic to *ace*/*hth, adhesion of calyx edges*/*hothead*) mutant that is characterized by a deficient fatty acid ω-alcohol dehydrogenase activity (Kurdyukov et al., 2006a). Increased accumulation of cell-wall-bound lipids and epicuticular waxes occurs in *bdg* (*bodyguard*) mutants compared to WT plants (Kurdyukov et al., 2006b). The cuticle of *lacs2* (*long-chain acyl-CoA synthetase*; Schnurr et al., 2004) an identical mutant as *bre1* (*Botrytis resistant*; Bessire et al., 2007) is thinner than that of WT plants and contains reduced amounts of dicarboxylic acid monomers in the cutin polyester. The *sma4* (*symptoms to multiple avr genotypes4*) is allelic to *lacs2* (Tang et al., 2007). The *fdh* (*fiddlehead*) is mutated in a gene encoding a protein involved in the synthesis of long-chain lipids (Yephremov et al., 1999; Pruitt et al., 2000; Voisin et al., 2009). The *pec1* (*permeable cuticle 1*) is characterized by a knockout of ATP BINDING CASSETTEG32 (ABCG32), an ABC transporter localized at the plasma membrane of epidermal cells; available evidence suggests that ABCG32 exports cutin precursors for the synthesis of the cuticular layer in the epidermal cell (Bessire et al., 2011). Abscisic acid (ABA) deficiency causes an increase cuticular permeability and resistance to *Botrytis cinerea* as observed in the *sitiens* as well as the *abi2* and *abi3* mutants of tomato and *Arabidopsis thaliana* respectively (Curvers et al., 2010; L’Haridon et al., 2011). An enhanced cuticular permeability and resistance to *Botrytis cinerea* was also observed in the *myb96-1* (*MYB96-deficient*) mutant characterized by downregulated ABA-dependent wax biosynthetic genes (Seo et al., 2011). In tomato, overexpression of SlSHINE3, a transcription factor expressed predominantly in the epidermis, leads to leaves with increased permeability, an increase in cutin monomer content and resistance to *Botrytis cinerea* and *Xanthomonas campestris pv. vesicatoria* (Buxdorf et al., 2014). An increase in resistance to *Botrytis cinerea* was observed when cutin monomers extracted from WT- and SlSHINE3-overexpressing leaves are applied to tomato leaves. Details on the amounts and quality of the cutin monomers or on their mode of action (direct versus indirect) that could explain this result are not known. In the same article, the authors show that only cutin monomers of SlSHINE3-overexpressing leaves induced the expression of defense genes in tomato (Buxdorf et al., 2014). But, not all mutants affected in the cuticle structure show an enhanced resistance to necrotrophic pathogens. The *cer1* mutant of *Arabidopsis thaliana* is affected in an enzyme predicted to be involved in alkane biosynthesis (Bourdenx et al., 2011). *CER1* shows the same expression pattern and localization as other enzymes expressed in the epidermis of aerial organs. Overexpression of *CER1* results in plants with a reduced permeability associated with an improved resistance to water deficient soils. Such plants showed a increased susceptibility to *Pseudomonas syringae pv. tomato* and to the necrotrophic *Sclerotinia sclerotiorum*. The *gl1* mutation affects cuticle formation, but is still susceptible to *Botrytis cinerea* (Xia et al., 2010; Benikhlef et al., 2013). The *rst1* (*RESURRECTION1*) mutant exhibits enhanced susceptibility to the biotrophic fungal pathogen *E. cichoracearum* but enhanced resistance to the necrotrophic fungal pathogens *Botrytis cinerea* and *Alternaria brassicicola*. RST1 is plasma membrane protein and is possibly involved in suppressing the biosynthesis of cuticle lipids; the increased levels of cutin monomers and cuticular waxes in *rst1* suggest this. Despite this, *rst1*shows a clear departure from the behavior of other mutants since the permeability of the cuticle is normal (Mang et al., 2009). Another intriguing observation was made with *Arabidopsis thaliana acp4* mutants defective in acyl carrier protein (ACP4). The *acp4* mutants were tested in the context of systemic acquired resistance (SAR); they are able to generate a mobile SAR signal from lower leaves inoculated with bacteria but unable to perceive it in the upper leaf. The *acp4* also display cuticular defects with reduced levels of fatty acids, alkanes and primary alcohols compared to WT plants associated with ultrastructural changes and an increased cuticular permeability (Xia et al., 2009). When wild type Col-0 plants were abraded to remove the cuticle in the upper leaves, SAR was also compromised. It was concluded that an intact cuticle is required for the onset of SAR. It remains difficult to explain how defects in the cuticle impart SAR. Abraded plants are not perfect mimics for the cuticle-defective *acp4* mutants and possibly other compensatory mechanisms might take place differently in both types of plants. It remains now to be shown how an intact cuticular layer can influence SAR. Soft mechanical stress (SMS) applied to leaves was shown to increase resistance to *Botrytis cinerea* and lead to the production of reactive oxygen species (ROS; Benikhlef et al., 2013). SMS resembles the delicate mechanical abrasion of the cuticle used by Xia et al. (2009) and it would now be interesting to know if abraded plants show increased resistance to *Botrytis cinerea*.

Considering the mutants listed in **Table 1**, modifications in cuticular structure associated with enhanced permeability are correlated with enhanced resistance to *Botrytis cinerea*. In addition to resistance, many of these mutants spontaneously accumulate ROS. For instance, the cuticular mutants *bdg* and *lacs2* constitutively produce a green fluorescence upon staining with 5-(and 6)-carboxy-29,79-dichloro dihydrofluorescein diacetate (DCF-DA) a fluorescent probe for ROS (L’Haridon et al., 2011; Benikhlef et al., 2013). Treatment of wild type leaf surfaces with fungal cutinase also results in ROS accumulation (L’Haridon et al., 2011). ROS has a multifaceted mode of action and can reach toxic levels acting directly as an antimicrobial or participate in various steps during the activation of defense responses such as modification of the cell wall, signal transduction pathways, programmed cell death, or post-translational regulation (De Tullio, 2010; Torres, 2010; Mittler et al., 2011). At this point, it is not well known why ROS are made in *bdg* and *lacs2* or in cutinase-treated leaves. Presumably, cutin monomers or other compounds accumulating in developmental mutants of the cuticle might be perceived by the plant and result in the production of ROS. A possible early event preceding ROS accumulation might be a Ca^2^^+^ burst as was shown after wounding or SMS (Beneloujaephajri et al., 2013; Benikhlef et al., 2013). ROS are produced earlier and in higher amounts after inoculation with *Botrytis cinerea* in the *aba2* and *aba3* mutants of ABA biosynthesis as well as in the wax biosynthesis mutant *myb96-1* and these plants were also shown to have an increased cuticular permeability (L’Haridon et al., 2011). All these examples offer the interesting possibility to find out how ROS are produced in relation to the cuticular properties.

CUTE, *lcr, hth, bdg, lacs2/bre1, sma4,* and *pec1* displayed increased resistance to *Botrytis cinerea* and the presence of a fungitoxic activity in leaf diffusates that correlated with an increased permeability of the cuticle (Bessire et al., 2007, 2011; Chassot et al., 2007). Thus, the presence of a fungitoxic activity appears to be mostly associated with an increase in cuticular permeability. The question now arises on the nature of the fungitoxic compound present in the leaf diffusates. At this point, it is tacitly assumed that in all cases the same compound is involved; a chemical characterization will eventually clarify this point. Another intriguing possibility is that phylloplane microbes might contribute directly or indirectly to this activity. For instance, the presence of distinct patterns of microbial communities was observed on the surface of different *Arabidopsis thaliana eceriferum* wax mutants (*cer1, cer6, cer9, cer16*) compared to the corresponding wild type ecotype *Ler* (Reisberg et al., 2013). This interesting observation shows that plant cuticular wax composition can affect the community composition of phyllosphere bacteria. Likely, it is possible that other changes in the composition of the plant surface might also affect bacterial communities. The extent to which such microbes contribute to the fungitoxic activities in leaf diffusates or even to fungal resistance is not known.

The pleiotropic syndrome exhibited in the cuticular mutants such as altered cuticle structure and deposition, altered chemical composition in cuticular lipids, organ fusions, changes in and cell and organ shape or resistance to pathogens suggest that plants adapt to the cuticular defects by compensatory mechanisms. To investigate such an adaptive compensatory mechanism a meta-analyses tool (MASTA; microarray overlap search tool and analysis) was developed and used for an *in-silico* analysis of gene expression profiles in hundreds of datasets (Voisin et al., 2009). This led to the identification of the *SERRATE* (*SE*) gene, which encodes a nuclear protein of RNA-processing multi-protein complexes, making it likely that small-RNA signaling is involved in the cuticular defect syndrome. The importance of the *SE* gene was confirmed with double mutants such as *lcr-se* and *bdg-se* that suppress the abnormal cuticle syndrome and resistance to *Botrytis cinerea*. These results support the hypothesis that various cuticular defects might induce a common signaling pathway that depends on the *SE* gene (Voisin et al., 2009). It will now be interesting to see if this type of analysis can be further used to identify aspects more specific to the fungal resistance response.

**FIGURE 1:**
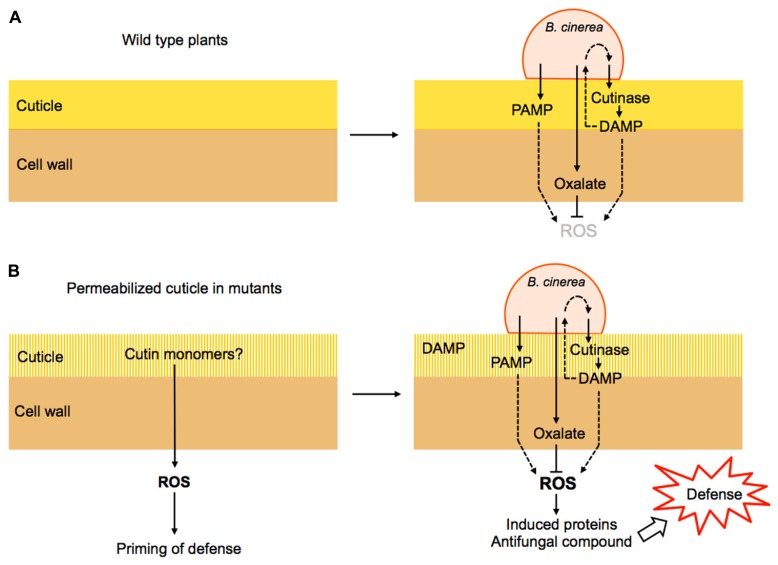
**Hypothetical model explaining cuticle-derived resistance to *B. cinerea*. (A)** During the infection of a wild type plant, *B. cinerea* releases cutinase and PAMPs that lead to its recognition and potentially to ROS formation and defense activation. However, the production of oxalate by *B. cinerea* interferes with ROS production and prevents efficient defenses thus allowing colonization. **(B)** In various mutants affected in the cuticle and its permeability (see **Table 1**), ROS are produced constitutively leading to the priming of defenses. Upon infection, fungal oxalate is insufficient to scavenge ROS and the plant defense is successful. The products of the activity of fungal cutinase are referred to as DAMPs that can also be perceived by the fungus and activate more cutinase expression.

The evidence provided by the effect of ectopic treatments with cutin monomers, overexpression of cutinase, ectopic treatments with cutinase and various cuticular mutants with increased permeability lead several scenarios that might explain the resistance of plants in relation to defective cuticles (Chassot et al., 2008). A permeable cuticle could involve a faster perception of putative products of the cuticle released upon the action of the cutinase. In addition, cuticle monomers might be over-produced in cuticular mutants from an incomplete cuticle polymer synthesis. The perception of such monomers would generate intracellular signals and trigger multifactorial defenses. The induced defenses might involve the production/release of ROS, antimicrobial proteins and of antifungal metabolites. A permeable cuticle might also allow a faster passage of potential elicitors from *Botrytis cinerea* or its inoculation medium through the epidermis wall into the cells where they might trigger a faster and more intensive defense reaction. The surprising potential for defense against *Botrytis cinerea* unveiled in CUTE plants and in the various cuticle mutants warrants further research to understand the molecular basis of this phenomenon (**Figure 1**).

A puzzling question concerns the full susceptibility of *Arabidopsis thaliana* to *Botrytis cinerea*. This is intriguing, since *Botrytis cinerea* releases cutinase and lipase during the penetration of leaves (Comménil et al., 1998) yet no resistance is visible. In contrast, our own experiments showed that when cutinase or lipase is applied on the surface of *Arabidopsis thaliana* leaves resistance and ROS are induced (Chassot et al., 2007; L’Haridon et al., 2011). Possibly, the timing or the quantity of enzymes produced by the fungus *in planta* is sufficient for penetration but not for inducing resistance. Alternatively, *Botrytis cinerea,* like other pathogens, might suppress induced defense responses in the plant. One possible suppressor could be oxalic acid, a know pathogenicity factor of *Botrytis cinerea* (Germeier et al., 1994; Pezet et al., 2004) and suppressor of ROS (Cessna et al., 2000). Several experimental lines support this hypothesis. For instance, biocontrol bacteria selected for their ability to metabolize oxalate can protect *Arabidopsis thaliana* against *Botrytis cinerea* (Schoonbeek et al., 2007). Also, transgenic plants overexpressing a fungal oxalate decarboxylase show an earlier and increased accumulation of ROS and an enhanced tolerance after inoculation with *Botrytis cinerea* (L’Haridon et al., 2011) or *Sclerotinia sclerotiorum* (Walz et al., 2008). This might explain why *Arabidopsis thaliana* is susceptible to *Botrytis cinerea,* despite the release of cutinase and lipase.

## FUTURE DIRECTIONS

How plants perceive changes in the level of cutin monomers is still not known and is a question that needs to be addressed. The experimental evidence accumulated so far makes it reasonable to assume that plants are equipped to perceive cutin monomers or other related products possibly by receptors. A genetic screening would be an approach of choice to identify such receptors. In fact, we are currently screening *Arabidopsis thaliana* mutants or ecotypes that lack an increase in resistance to *Botrytis cinerea* after treatment with fungal cutinase. A series of mutants and ecotypes could be identified, all displaying an increased in susceptibility to *Botrytis cinerea.* These results are now being followed up; one predicts that such mutants could be blocked in either a putative receptor for cutinase-generated monomers or alternatively in any step downstream of it.

Using the available genome-wide gene expression microarray data, one can identify common genetic elements during the resistance syndrome in cuticle deficient mutants. Using the MASTA (Reina-Pinto et al., 2009b), differentially expressed gene lists can be generated and classified according to the gene ontology (GO). Using this strategy a list of 25 upregulated genes statistically significant under the GO category “response to fungus” can be identified. These genes point toward common functions that might relate to the resistance syndrome in cuticle deficient mutants and they deserve further attention.

Another intriguing question is the chemical nature of the fungitoxicity in the diffusates of cuticular mutants. It is not clear whether the same chemical causes the observed activity for each mutant; a bioassay-assisted chemical identification is under way to clarify this point.

## Conflict of Interest Statement

The authors declare that the research was conducted in the absence of any commercial or financial relationships that could be construed as a potential conflict of interest.
